# Long noncoding RNA mediates enzalutamide resistance and transformation in neuroendocrine prostate cancer

**DOI:** 10.3389/fonc.2024.1481777

**Published:** 2024-11-25

**Authors:** Zhe Zhu, Wenjing Xuan, Chaohui Wang, Chancan Li

**Affiliations:** ^1^ Department of Urology, Anhui No.2 Provincial People’s Hospital, HeFei, China; ^2^ Department of Obstetrics, Anhui No.2 Provincial People’s Hospital, HeFei, China; ^3^ Department of Thyroid and Breast Surgery, Affiliated Hospital of Jiangsu University, Zhenjiang, China

**Keywords:** long noncoding RNA, enzalutamide, enzalutamide resistance, neuroendocrine prostate cancer, AR, prostate cancer

## Abstract

Prostate cancer is a malignant tumor caused by the malignant proliferation of epithelial cells, which is highly heterogeneous and drug-resistant, and neuroendocrine prostate cancer (NEPC) is an essential cause of drug resistance in its late stage. Elucidating the evolution of NEPC and the resistance process of enzalutamide, a novel antiandrogen, will be of great help in improving the prognosis of patients. As a research hotspot in the field of molecular biology in recent years, the wide range of biological functions of long noncoding RNAs (lncRNAs) has demonstrated their position in the therapeutic process of many diseases, and a large number of studies have revealed their critical roles in tumor progression and drug resistance. Therefore, elucidating the involvement of lncRNAs in the formation of NEPCs and their interrelationship with enzalutamide resistance may provide new ideas for a deeper understanding of the development of this disease and the occurrence of enzalutamide resistance and give a new direction for reversing the therapeutic dilemma of advanced prostate cancer. This article focuses on lncRNAs that regulate enzalutamide resistance and the neuroendocrine transition of prostate cancer through epigenetic, androgen receptor (AR) signaling, and non-AR pathways that act as “molecular sponges” interacting with miRNAs. Some insights into these mechanisms are used to provide some help for subsequent research in this area.

## 1 Background

Prostate cancer progresses slowly but with a relatively high degree of malignancy, new cases with late clinical staging, high Gleason grade, and clinical presentation of prostate cancer with multiple metastases, which have lost the chance of surgery (1). Androgen deprivation therapy (ADT) is the clinically preferred treatment for prostate cancer and significantly inhibits prostate cancer progression, but almost all patients progress to the more aggressive castration-resistant prostate cancer (CRPC) after 12-18 months of treatment (2). The emergence of a new generation of potent androgen receptor inhibitors, represented by enzalutamide, has revolutionized the concept of novel endocrine therapy for CRPC and has also primarily prolonged the survival time of CRPC-resistant patients. However, patients with effective initial therapy still experience drug resistance after a remission period of approximately 11.2 months (3). The transformation from adenocarcinoma to NEPC that occurs in patients with CRPC is now considered to be a significant cause of disease progression. Primary prostate neuroendocrine tumors are extremely rare, accounting for approximately 0.5%-2% of all prostate tumors, but the incidence of this tumor rises significantly after endocrine therapy, with 17%-30% of prostate cancer patients presenting with NEPC after endocrine therapy (4, 5). lncRNAs have essential biological functions in cells and can regulate relevant gene expression in epigenetic, pre-transcriptional and post-transcriptional processes, which are further involved in tumor development, metastasis, and drug resistance (6, 7). This article details how lncRNAs mediate CRPC treatment resistance to enzalutamide and its conversion to neuroendocrine.

## 2 Clinical and molecular features and evolutionary mechanisms of neuroendocrine prostate cancer

**Figure 1:**
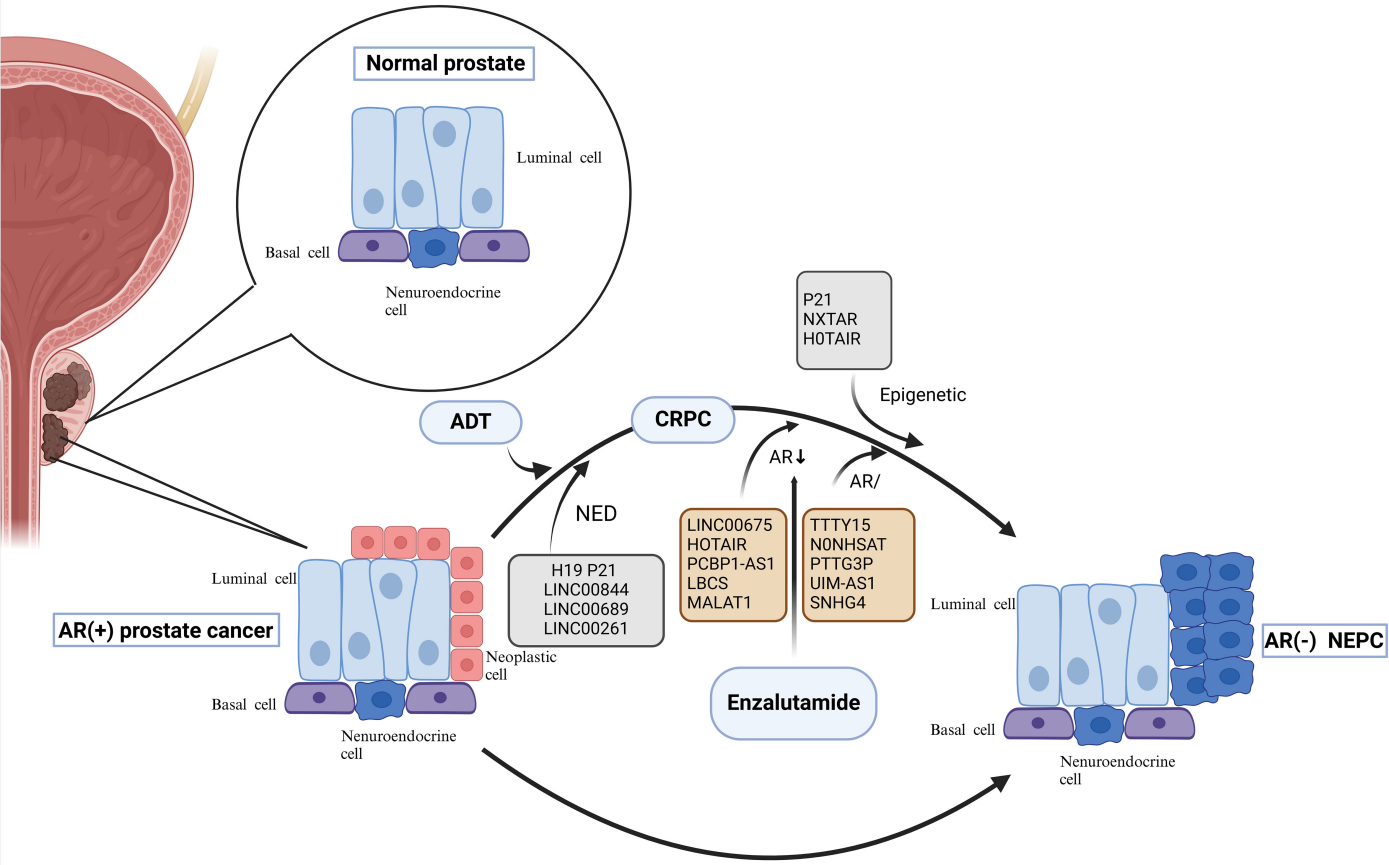
Neuroendocrine Evolution and drug resistance mechanism of prostate cancer. Evolution of NEPC. On the one hand, under the pressure of endocrine therapy, adenocarcinoma cells with reduced or no AR expression undergo neuroendocrine transformation; on the other hand, neuroendocrine cells in the prostate gland that share a common origin with basal and official cells progress directly to NEPC. Among them, lncRNAs can participate in the transformation of prostate cancer to NEPC by regulating the NED process; lnc RNAs can also mediate the resistance process of enzalutamide through the epigenetic pathway, the AR pathway, and related non-AR pathways. AR, androgen receptor; ADT, androgen deprivation therapy; NED, neuroendocrine differentiation; CRPC, castration-resistant prostate cancer; NEPC, neuroendocrine prostate cancer; lncRNAs, Long noncoding RNAs.

NEPC is a lethal subtype of prostate cancer that encompasses all phenotypes ranging from prostate adenocarcinoma with focal NEPC cells to pure small-cell neuroendocrine carcinoma. Prostate tissue mainly contains ductal cells, basal cells, and neuroendocrine cells (1%) located in the basal layer, which secrete proteins involved in the composition of male semen (8, 9). NEPC can be divided into primary and treatment-related neuroendocrine prostatic carcinoma (t-NEPC); the former refers to NEPC that is present at the time of tumor development and accounts for about 0.5%-2% of first-diagnosed prostate cancers, while the latter refers to prostate cancers that show partial or complete neuroendocrine differentiation after ADT, which accounts for about 10%-20% of CRPC (10, 11). Primary NEPC is relatively rare and is clinically characterized by susceptibility to visceral metastases, low prostate-specific antigen (PSA) levels, and mutations or deletions in the TP53 and RB1 genes (12). Histologically, t-NEPC may present as a neuroendocrine carcinoma or a mixture of neuroendocrine carcinoma and adenocarcinoma (13, 14). Compared with adenocarcinoma, NEPC has unique gene expression characteristics. NEPC cells have delicate chromatin in the nucleus, whereas adenocarcinoma cells have a more obvious nucleolus, which suggests that the distribution of heterochromatin in NEPC is quite different from that in adenocarcinoma. In addition, it was found that there are neurodevelopment-related transcription factors such as MYCN, FOXA2, BRN2, ASCL1, and NEUROD1 that activate and drive the development of NEPC (15–17). For the evolution of NEPC (**Figure 1**), many previous studies have focused on the neuroendocrine transformation of prostate adenocarcinoma cells under the pressure of endocrine therapy, with reduced or loss of AR expression and expression of neuroendocrine markers such as synaptophysin (SYP), chromogranin A (CgA), and neuron-specific enolase (NSE), thereby transdifferentiating into NEPC (18–20). However, recent studies have found that NEPC may also originate from neuroendocrine cells with a neuroendocrine phenotype already present in adenocarcinomas. A scRNA-seq-based research found that a subpopulation of CRPC-like cells and a subpopulation of neuroendocrine cells independent of the AR signaling pathway, which share the exact origin as prostate luminal and basal cells, were present in primary prostate cancer tissues without endocrine therapy. ADT resulted in the predominance of these more value-adding AR-positive neuroendocrine cells, leading to rapid resistance to endocrine therapy and the development of NEPC in prostate cancer (18, 21, 22). NEPC is highly aggressive and lethal, capable of extensive metastasis to organs and bones, and has limited treatment options, insensitivity to hormonal therapy, and short-lived effects of chemotherapy, with a median survival of about seven months from diagnosis. It represents the terminal stage of prostate cancer (23, 24). The relevant literature suggests that the availability of the more potent hormone enzalutamide may increase the incidence of NEPC (25). Diagnosing NEPC in clinical practice is also tricky. It usually relies on features such as the paradoxical decrease in PSA and tumor metastasis; therefore, exploring its diagnostic markers and therapeutic targets and determining its resistance mechanism are the primary needs of clinical diagnosis and treatment.

## 3 Molecular characterization of lncRNAs

lncRNA is a class of RNA molecules with more than 200 bp length. lncRNAs are expressed less than mRNAs and are involved in tumorigenesis, drug resistance, metastasis, and prognosis by interacting with DNA, RNA, and proteins in pre-transcriptional and post-transcriptional processes (26, 27). lncRNA alterations are essential drivers of tumorigenesis, progression, and metastasis. They are involved in tumor progression through mechanisms such as chromatin remodeling, transcriptional co-activation or repression, regulation of protein activity, post-transcriptional regulation, or acting as a deceptive element (19, 28, 29). The development of molecular detection techniques has led to a significant increase in the detection of lncRNAs in related fields, such as the detection of the prostate cancer lncRNA-associated transcript prostate cancer associated transcript (PCAT), paved the way for many subsequent studies. Another pan-cancer study, MiTranscriptome, identified various tissue and tumor type-specific lncRNAs (30, 31). One study investigated the expression of LINC00467 in prostate cancer tissues and cells using Western blot analysis and reverse-transcriptase PCR (RT-qPCR), and they determined that the expression of LINC00467 was upregulated in prostate cancer tissues and cells. Western blot analysis showed that LINC00467 could regulate the STAT3 pathway, and bioinformatics analysis and salvage experiments indicated that LINC00467 promoted prostate cancer progression and mediated the transformation of prostate cancer to NEPC through the miR-494-3p/STAT3 axis (32). In addition, researchers have found that one of the most prominent mechanisms by which Linc00467 regulates the development of other human malignant tumors is the Linc00467/miRNA/mRNA signaling regulation axis (**Table 1**). Here, LncRNAs have the properties of ceRNAs and mediate tumorigenesis and development by interacting with miRNAs. Specifically, it was found in hepatocellular carcinoma cells that Linc00467 could exert its pro-tumorigenic effect by adsorbing and inhibiting the expression of miR-9-5P/miR-18a-5p and deregulating the inhibitory effect of miR-9-5p/miR-18a-5p on PPARA/NEDD9 (33). Linc00467 can target miR-4779 and miR-7978 in lung cancer to promote lung cancer cell proliferation, invasion and metastasis, and it can also adsorb miR-20b-5p to relieve the inhibitory effect of miR-20b-5p on CCND1 to promote lung adenocarcinoma cell growth (34, 35). In the study of cervical cancer, Linc00467 exerted its pro-cancer effect by targeting miR-107 and promoting the expression of KIF23 (36). In fact, in colorectal cancer, neurological tumors, and head and neck phosphoribocytic carcinoma, Linc00467 can exert its pro-cancer effects by acting on miRNAs with the properties of ceRNAs (37–39). In prostate cancer, it has been shown that Linc00467 can promote the expression of STAT3 by acting on miR-494-3, which in turn promotes the malignant progression of prostate cancer and its transformation to NEPC (32). In recent years, some researchers have discovered differential expression of lncRNAs in tumor drug-resistant cells through relevant technologies. For example, Yang (40) used high-throughput lncRNA expression profiling microarrays to detect lung cancer cell lines and cisplatin-resistant cell lines and found 1380 differentially expressed lncRNAs. Similarly, Jiang (41) used high-throughput lncRNA expression profiling microarrays to detect differentially expressed lncRNAs in breast cancer cell lines and adriamycin-resistant cell lines, and found that 1649 lncRNAs were up-regulated and 1267 lncRNAs were down-regulated. In addition, researchers have utilized lncRNA microarrays to detect differentially expressed lncRNAs and mRNAs in nasopharyngeal carcinoma and found that the expression of 3 lncRNAs and 46 mRNAs were significantly correlated (42). As a gene regulatory mechanism, ceRNAs can indirectly participate in the post-transcriptional regulation of mRNAs by competitively binding to the response elements of miRNAs. lncRNAs can play a regulatory role post-transcriptionally in the form of lncRNA-miRNAs. As mentioned above, lncRNAs act as miRNA “iRNAsned. spongese that influence the proteome by mediating complex formation. It was found that miRNAs interacting with the 3’ untranslated region (UTR) of target mRNAs can exert inhibitory effects on both RNAs and proteins. They also noted that the 3’ UTR of ceRNAs is shortened, which regulates RNA transcripts at the level of other RNA transcripts by competing for shared miRNAs and ultimately contributes to tumorigenesis. this concept of ceRNAs can also be applied to the interactions of lncRNAs and miRNAs depending on whether their genes play a promotional or inhibitory role in tumor development (43, 44). The specific signaling mechanisms by which lncRNAs interact with miRNAs to mediate neuroendocrine transformation in prostate cancer and enzalutamide resistance will also be developed later.

**Table 1:** Linc00467/miRNA/mRNA signaling axis regulates multiple tumor progression outcomes.

Tumor type	Mechanism of Linc00467 participation	Outcome	Reference
Liver cancer	Deregulation of PPARA/NEDD9 by inhibition of miR-9-5P/miR-18a-5p expression	promote	(33)
Lung cancer	Targeting miR-4779, miR-7978; adsorption of miR-20b-5p and derepression of CCND1 by miR-20b-5p	promote	(34, 35)
Cervical carcinoma	Targeting miR-107 to promote KIF23 expression	promote	(36)
Prostatic cancer	Acting on miR-494-3 promotes STAT3 expression	promote	(32)

PPARA, Peroxisome Proliferator-Activated Receptor-Alpha; NEDD9, Neural Precursor Cell Expressed, Developmentally Down-regulated 9; CCND1, Cyclin D1 is a Protein Coding gene; KIF23, Kinesin Family Member 23; STAT3, Signal Transducer and Activator of Transcription 3.

## 4 LncRNAs mediate the transformation of prostate cancer to NEPC

### 4.1 LncRNAs regulate neuroendocrine in prostate cancer through the AR signaling pathway

AR is a central signaling pathway in prostate cancer. Androgens bind to membrane-localized AR and activate AR, which binds to its homologous response elements, thereby recruiting co-regulatory factors to promote the expression of relevant genes and ultimately promote tumor cell proliferation, malignant metastasis, and resistance to relevant chemotherapeutic agents (45). Although therapeutic approaches to develop inhibitors against AR are widely used in the clinic, AR signaling can also be initiated in prostate cancer cells from the bypass via glucocorticoid receptor (GR) signaling, thus making prostate cancer cells resistant to AR inhibitors (46). Improving AR resistance is currently the most important part of prostate cancer research that needs to be addressed, and the development of lncRNA studies related to the AR signaling pathway may help to overcome AR resistance in prostate cancer. AR resistance is a great challenge to be faced during the current prostate cancer treatment, and there are multiple lncRNAs involved in the gradual development of AR resistance in prostate cancer, which can play the role of pro- or oncogenes. Long Noncoding RNA H19 (lncRNA H19) is significantly overexpressed in NEPC patients. It was confirmed that H19 induced the differentiation of prostate cancer to NEPC and increased the resistance of tumor cells to ADT. It was shown that H19 acts as an epigenetic regulator in NEPC and regulates histone H3 lysine trimethylation at position 27 (H3K27me3) and histone H3 lysine trimethylation at position 4 (H3K4me3) by binding to the polycomb repressive complex 2 (PRC2) complex. This remodeled AR signaling (ARS) and NE genes in the vicinity of chromatin, which in turn regulated the expression of ARS and NE genes and activated the AR signaling pathway, ultimately promoting the transformation of prostate cancer to NEPC (23). Other studies have shown that lncRNAs can regulate enzalutamide resistance by promoting neuroendocrine in prostate cancer cells.

Luo’s study showed that lncRNA-p21 increases neuroendocrine differentiation of prostate cancer induced by enzalutamide treatment. Enhancer of zeste homolog 2 (EZH2) is the core unit of the epigenetic effector PRC2, and lncRNA-p21 binds to EZH2 while inhibiting the binding of EZH2 to another pair of lncRNAs that have stabilizing effects on PRC2, thereby decreasing interactions between the core subunits of PRC2, interfering with the PRC2 formation and enhance the methyltransferase activity of EZH2. LncRNA-p21 also promotes the interaction of EZH2 with ATK and ATAT3, exerts multiple functions in this pathway, enhances STAT3 methylation, and induces NED development and drug resistance (47, 48). lncRNAs not only act as oncogenes to promote prostate cancer progression but also suppress the malignant phenotype of this tumor by inhibiting the AR signaling pathway. Shreyas (49) showed that LINC00844 has an inhibitory effect on tumor progression and metastasis, and the expression level of LINC00844 in normal prostate tissues was much higher than that of metastatic prostate cancer in clinical specimens. LINC00844 has an inverse role in AR signaling and represses global transcription of androgen-regulated genes. In this process, LINC00844 promotes the expression of N-myc downstream regulatory gene 1 (NDRG1), and NDRG1 acts as an AR repressor to inhibit AR expression, and the metastasis and differentiation of prostate cancer cells are also inhibited after the AR signaling pathway is suppressed. However, many related literatures indicate that lncRNAs can affect the malignant phenotype of prostate cancer through the AR signaling pathway. However, the compensatory effect of the AR signaling pathway through bypass signaling leads to poor efficacy and a high recurrence rate of prostate cancer treatment relying on the AR pathway, which also indicates that AR signaling pathway-associated lncRNAs that can be used as efficient prostate cancer therapeutic targets have yet to be explored.

### 4.2 LncRNAs regulate neuroendocrine in prostate cancer through non-AR signaling pathways

**Figure 2:**
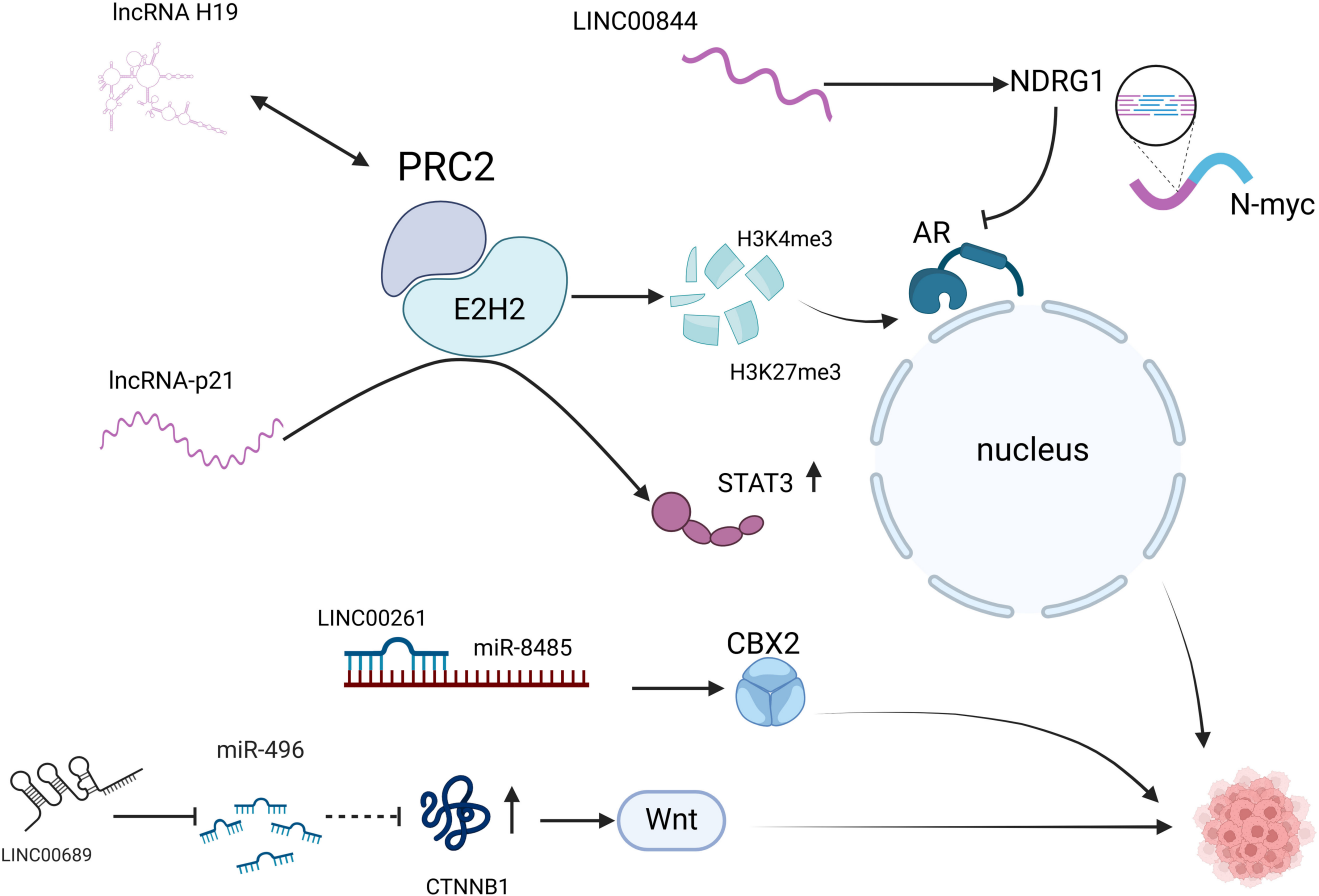
LncRNAs are involved in the neuroendocrine secretion of prostate cancer. AR pathway: lncRNA H19 activates AR signaling by binding to the PRC2 complex and regulating H3K27me3 and H3K4me3; lncRNA-p21 binds to EZH2 while inhibiting the binding of EZH2 to HOTAIR, interfering with PRC2 formation, and enhances methylation of STAT3; LINC00844: promotes NDRG1 expression and inhibits AR expression. Non-AR pathway: LINC00689 promotes CTNNB1 expression and activates the Wnt signaling pathway; LINC00261 acts as a miR-8485 molecular sponge and enhances CBX2 activity. AR, androgen receptor; PRC2, polycomb repressive complex 2; H3K27me3, Trimethylation modification of lysine at position 27 of histone H3; H3K4me3, The fourth lysine of histone H3 undergoes trimethylation catalyzed by methyltransferase (HMT); EZH2, enhancer of zeste homolog 2; STAT3, signal transducer and activator of transcription 3, STAT3; NDRG1, N-myc downstream regulatory gene 1; CTNNB1, Catenin Beta 1 is a Protein Coding gene; CBX2, chromobox homolog2.

Wnt signaling is a class of evolutionarily conserved signal transduction cascade pathways that play a central role in embryogenesis, trauma repair, and malignancy (50, 51). Wnts can activate various intracellular pathways, including the classical Wnt/β-catenin and non-classical Wnt pathways. Classical Wnt/β-catenin signaling is evolutionarily highly conserved and is the most frequent Wnt pathway in prostate cancer (52, 53). Meng (54)found that the expression of LINC00689 was significantly elevated in advanced prostate cancer cells, and thus, knockdown of LINC00689 significantly inhibited the proliferation, invasion, and further differentiation of this tumor and ultimately induced apoptosis of prostate cancer cells. Additional mechanistic studies showed that LINC00689 acted as a ceRNA for the calmodulin-associated protein CTNNB1 bound to miR-496 upstream of CTNNB1 and inhibited the expression of the miRNA. miR-496 inhibited the inhibitory effect on CTNNB1, and the expression of CTNNB1 was increased, activating the Wnt signaling pathway to promote proliferation, invasion, and differentiation. This Study illustrates that LINC00689 promotes prostate cancer progression through activation of the Wnt pathway via the miR-496/CTN-NB1 axis, and thus, studying LINC0068 may provide a new direction for further treatment of prostate cancer. Furthermore, In addition to the Wnt signaling pathway, it was shown that the LncRNA LINC00261, which is highly conserved, is also involved in regulating prostate cancer transformation to NEPC. LINC00261 is significantly upregulated in patients with NEPC and acts as a sponge for the miR-8485 molecule in the cytoplasm to increase the activity of chromobox homolog2 (CBX2), thereby promoting the transformation of NEPC (55). In summary, lncRNAs can mediate prostate conversion to NEPC through the AR signaling pathway, the Wnt signaling pathway, and interaction with mRNAs (**Figure 2**).

## 5 LncRNAs mediate enzalutamide resistance in prostate cancer

### 5.1 LncRNAs are involved in epigenetic modifications to regulate enzalutamide resistance

Epigenetic modifications are closely related to tumorigenesis and development, mainly through DNA methylation, histone demethylation, and other ways to regulate gene function and expression levels. Abnormal DNA methylation is one of the critical epigenetic modifications that drive the occurrence and development of cancer. GHILDIYAL (56) reported for the first time that lncRNA-NXTAR (LOC105373241) is located on chromosome Xq12 and is repressed for expression in prostate tumors. NXTAR enhances cellular resistance to enzalutamide by interacting with AR at the epigenetic level. It was noted that NXTAR, by binding upstream of the AR promoter, promotes H3K27 methylation at the epigenetic level by mediating (enhancer of zeste homolog 2) EZH2 recruitment, resulting in a significant reduction or loss of AR and AR-V7 expression.

Conversely, AR can bind to the NXTAR promoter and inhibit AR expression using a small molecule inhibitor of ACK1/TNK2, thereby inhibiting the proliferation of enzalutamide-resistant cells and reducing enzalutamide resistance during tumor therapy. It suggests that this pharmacological restoration approach’s up-regulation of NXTAR expression can provide new ideas for treating patients who develop resistance to new-generation AR antagonists.

### 5.2 Involvement of lncRNAs in the AR signaling pathway regulates enzalutamide resistance

**Figure 3:**
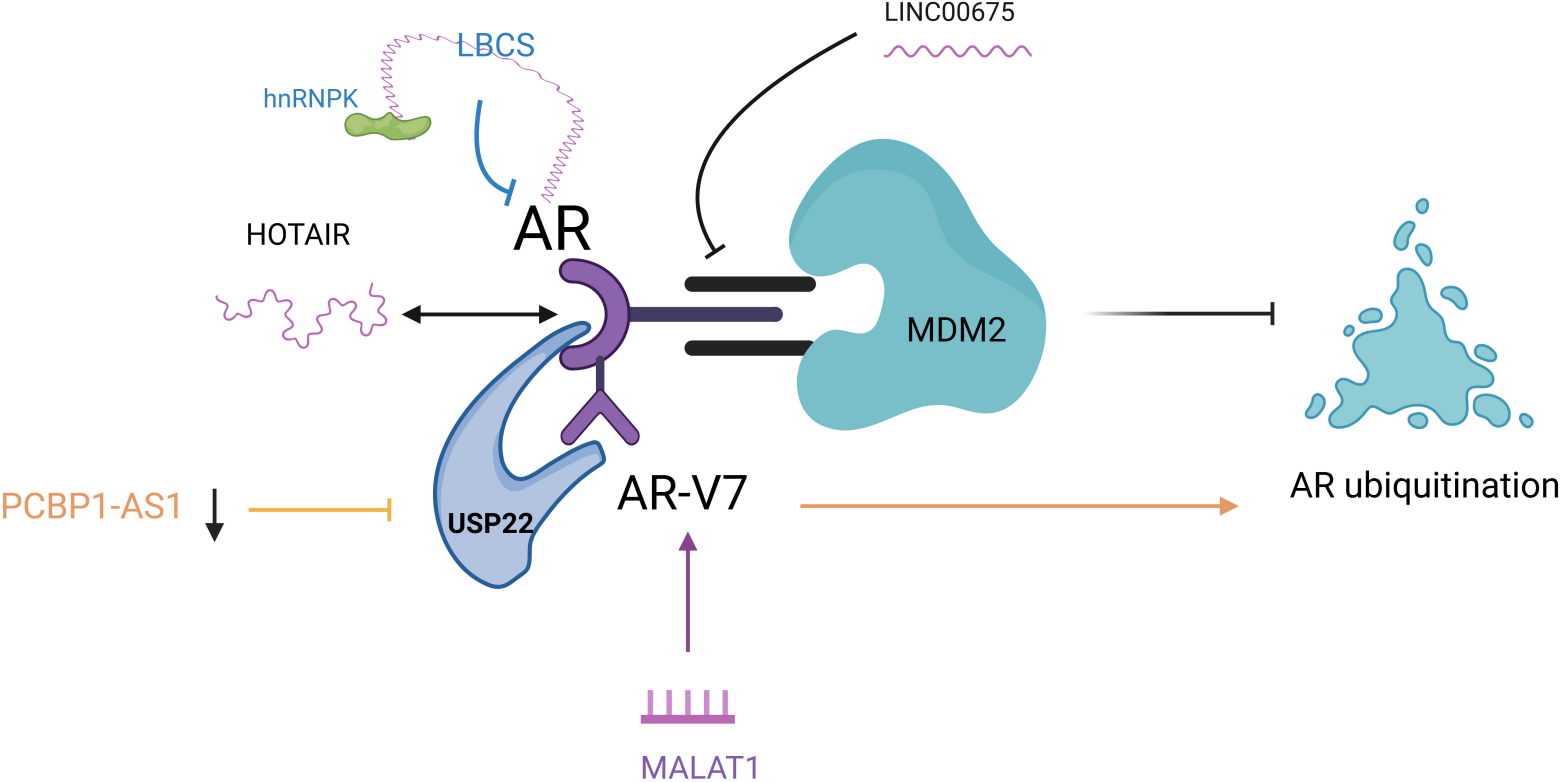
LncRNAs are involved in the AR pathway to regulate enzalutamide resistance. LINC00675: blocked the inter-AR and MDM2 region; HOTAIR: HOTAIR binds to AR to block its interaction with MDM2 and inhibit AR ubiquitination; PCBP1-AS1: PCBP1-AS1 expression was reduced to inhibit AR/AR-V7 and USP22 binding and promote AR ubiquitination; LBCS: acted as a scaffold for interaction with hnRNPK and AR mRNA interaction to inhibit AR expression; MALAT1: induced high expression of MALAT1/AR-V7 axis. AR, androgen receptor; MDM2, mousedouble minute 2; AR/AR-V7, Androgen receptor splicing variant 7; USP22, ubiquitin-specific processing peptidase 22; hnRNPK, heterogeneous nuclear ribonucleoprotein; MALAT1, metastasis associated lung adenocarcinoma transcript 1.

AR is a central signaling pathway in prostate cancer. lncRNAs can also affect enzalutamide resistance by regulating AR signaling axis transduction through different mechanisms (**Figure 3**). LINC00675 promotes depot resistance by blocking the binding region between AR and MDM2 and protects against ubiquitination-mediated degradation. Meanwhile, antisense oligonucleotides (ASO)-LINC00675 significantly inhibited enzalutamide resistance in CRPC cells (57). lncRNA-HOTAIR from the HOXC genome is significantly increased in prostate cancer cell lines, and HOTAIR binds to AR proteins to block their interaction with the E3 ubiquitin ligase MDM2, thereby preventing AR ubiquitination and protein degradation. Knockdown of HOTAIR inhibits the proliferation of enzalutamide-resistant tumor cells and is a potential therapeutic target for reversing enzalutamide resistance (26, 58). CHANG’s (59) study concluded that HOTAIR is involved in NEPC development and drug resistance through multiple mechanisms. HOTAIR is a downstream target RNA of the neuronal restriction silencing element REST, which plays a central role in NEPC. *In vitro* experiments confirmed that HOTAIR was significantly upregulated in CRPC cell lines with neuroendocrine (NE) phenotype and synchronized with the increased expression of trans-neuroendocrine markers. Knockdown of the HOTAIR gene resulted in the suppression of cytokine 6 (IL-6)-induced NED, which led to the hypothesis that HOTAIR is involved in regulating IL-6-induced NED process. In addition, a gene ontology analysis of dysregulated genes in CRPC cell lines overexpressing HOTAIR identified the autophagy pathway. The autophagy pathway has a crucial role in IL-6-induced NED and chemoresistance in NEPC, and HOTAIR has demonstrated that HOTAIR may have a regulatory role in the autophagy pathway in developing NEPC and drug resistance (60).

Zhang (61) was the first to illustrate the vital role of lncRNA PCBP1-AS1 in CRPC resistance. They found that both PCBP1-AS1 and ubiquitin-specific processing peptidase 22 (USP22) could bind to the NTD of androgen receptor shear variant 7 (AR/AR-V7) region and interfere with PCBP1-AS1 in cells significantly attenuated the binding ability of AR/AR-V7 and USP22. At the same time, AR ubiquitination levels were significantly increased, thereby enhancing the sensitivity of C4-2EnzR cells to Enzalutamide. This Study demonstrated that targeting PCBP1-AS1 increases the sensitivity of prostate cancer cells to Enzalutamide by reducing the binding of USP22 to AR-V7 or AR and decreasing the stability of the complex, providing new ideas for the clinical treatment of drug-resistant patients with CRPC. LncRNA-LBCS was significantly down-regulated in CRPC, and mechanistic studies revealed that LBCs can act as a scaffold to interact with RNA-binding protein hnRNPK and AR mRNA to form a complex as a way to inhibit AR expression (62). WANG (63) validated the role of metastasis-associated lung adenocarcinoma transcript 1 (MALAT1) in enzalutamide resistance using enzalutamide-resistant cell lines. It demonstrated that enzalutamide promotes the development of resistance by inducing high expression of the MALAT1/AR-V7 axis and that, in contrast, targeting the MALAT1/AR-V7 axis could restore the sensitivity of resistant cells to enzalutamide treatment.

### 5.3 LncRNAs regulate enzalutamide resistance through non-AR transduction pathways

In addition to their involvement in the AR signaling pathway, lncRNAs can regulate enzalutamide resistance accordingly through interactions with miRNAs and positive and negative feedback (**Table 2**). LncRNA TTTY15 from the Y chromosome regulates drug resistance in prostate cancer cells by upregulating cell division protein kinase 6 (cell division protein kinase 6) and fibronectin-1 (fibronectin-1) expression via sponge adsorption of miRNA-let-7 (64). A study by Chen (65) indicated that enzalutamide resistance-associated lncRNA NONHSAT-210528 can function as a competitive endogenous RNA (ceRNA) that promotes prostate cancer cell invasion and drug resistance through the miR-21-5p/YOD1 signaling pathway. They noted that NONHSAT-210528 promoted the expression of YOD1, an element involved in the regulation of enzalutamide resistance, by participating in the regulation of miR-21-5p expression in enzalutamide-resistant prostate cancer cells and that when the miR-21-5p expression was inhibited, the invasive effect of LncRNA-NONHSAT-210528 on enzalutamide-resistant cells was significantly reduced. LncRNA PTTG3P can upregulate the expression of Pituitary tumor -transforming gene-1 (PTTG1) by competitive binding to miR-146a-3p, thereby promoting the development of enzalutamide resistance during prostate malignancy treatment (66). With the development of sequencing technologies, there is increasing evidence that novel lncRNAs are involved in human tumorigenesis and progression (67, 68). SHI (69) identified VIM-AS1 as a key LncRNA regulator; VIM-AS1 overexpression reduced the sensitivity to enzalutamide treatment, which plays a regulatory role in prostate cancer proliferation as well as enzalutamide sensitivity through the VIM-AS1/IGF2BP2/HMGCS1 axis. They showed that VIM-AS1 promotes the mRNA stability of 3-hydroxy-3-methylglutaryl-CoA synthase 1 (HMGCS1) by interacting with growth factor 2 mRNA binding protein 2 (IGF2BP2), which enhances HMGCS1 expression.

**Table 2:** Involvement of lncRNAs in non-AR pathways to regulate enzalutamide resistance.

LncRNAs	Pathway	Enzalutamide Resistance Outcome	Reference
TTTY15	Molecular sponge adsorption of miRNA-let-7	promote	(64)
NONHSAT	miR-21-5p/YOD1 signaling pathway	promote	(65)
PTTG3P	Competitive binding to miR-146a-3p upregulates PTTG1	promote	(66)
VIM-AS1	VIM-AS1/IGF2BP2/HMGCS1 Axis	promote	(69)
SNHG4	let-7a/RREB1 positive feedback and ceRNA network	promote	(70–72)

miRNA-let-7, A non coding microRNA called let-7; miR-21-5p, One of the members of the microRNA family; YOD1, Ubiquitin thioesterase OTU1; miR-146a-3p, One of the members of the microRNA family; PTTG1, Pituitary tumor-transforming gene-1; VIM-AS1, Long non coding RNA vimentin antisense RNA1; IGF2BP2, growth factor 2 mRNA binding protein 2; HMGCS1, 3-hydroxy-3-methylglutaryl-CoA synthase 1; RREB1, ras responsive element binding protein 1; ceRNA, competing endogenous RNAs.

Conversely, the knockdown of HMGCS1 expression ameliorated VIM-AS1 overexpression-induced prostate tumor cell progression and enzalutamide resistance. It was found that SNHG4, as one of the members of the SNHG family, belongs to the lncRNA subgroup, which is involved in the regulation of various biological expression processes in humans, such as transcription and translation of genes and modification of RNAs and proteins (70, 71). Dong (72) indicated that SNHG4 promotes prostate tumor cell growth and drug resistance through let-7a/RREB1 positive feedback and ceRNA network. The specific mechanism is that SNHG4 regulates the cell cycle control factors RRM2, EZH2, AURKA, and TK1 through a let-7 miRNA-mediated ceRNA regulatory network, which regulates gene expression, apoptosis, and tumor cell proliferation and enzalutamide resistance. In addition, RREB1 activates SNHG4 transcription and is regulated by the SNHG 4/let-7/RREB one feedback loop.

## 6 Involvement of lncRNAs in relevant therapeutic targets for prostate cancer

The ideal situation for tumor treatment is to kill tumor cells precisely and effectively without damaging normal cells. Early-stage limited prostate cancer can be cured by radical surgery or radiotherapy, while advanced prostate cancer is mainly treated with androgen deprivation therapy (ADT)-based combination therapy, but also gradually develops drug resistance and a neuroendocrine transformation. The current first-line treatment regimen for NEPC is a platinum-based chemotherapy regimen, but the results are poor. Targeted therapy based on small molecule inhibitors has made some breakthroughs in clinical trials for NEPC. NEPC highly expresses the oncogenic transcription factor MYCN and the cell cycle kinase AURK, and the small molecule inhibitor alisertib, which targets MYCN-AURKA, has achieved some efficacy in phase II clinical trials (73). Han (16) identified significant activation of the FOXA2-KIT signaling axis in t-NEPC, identifying the KIT signaling pathway as a therapeutic target for NEPC, but current clinical trials of KIT inhibitors in prostate cancer have not reached valid endpoints (phase III) (74). In addition, DLL3/CD3 dual-antibody Tarlatamab/AMG757 is effective in treating small-cell lung cancer (75, 76). Tarlatamab/AMG757 promotes immune response and immunotherapy by bidirectionally targeting DLL3-expressing neuroendocrine cancer cells and CD3-positive cytotoxic T cells (77). It has been shown that NEPC cells highly express DLL3, and DLL3 can sensitize the therapeutic effect of AMG757 in the t-NEPC model (78). This study provides preclinical basic research for the clinical application of AMG757 in NEPC, which is now in phase I clinical trials. lncRNAs are transcription products similar to most mRNAs, and they serve as targets for small interfering RNAs (siRNAs) and small molecule inhibitors (79). This targeting of lncRNAs based on the use of siRNAs is very successful *in vitro*, but *in vivo*, the major challenge is targeting tumors with low efficiency and poor stability. However, *in vivo*, we found lncRNA-HOTAIR, which can act precisely on cancer cells using peptide nucleic acids (PNAs) bound to pH-(Low) Insertion Peptides (pHLIP) and inhibited the interaction of HOTAIR with EZH2. This also overcomes the challenges faced by *in vivo* siRNA technology (80).

Ongoing clinical trials of LncRNA-targeted therapies in prostate cancer are still minimal, which is a direction we need to explore further in the future (**Table 3**). We found that SSTR 5-AS1 is one of the solid predictive markers of t-NEPC. SSTR 5-AS1 has been extensively studied in neuroendocrine tumors of non-prostate origin with significant clinical potential (81–84). SSTR is used in the clinic for tumor imaging (85, 86), as a predictive marker for the prognosis of relevant tumors (87), and as a target involved in the course of therapy (82). A phase I clinical trial of SSTR 5 in patients with metastatic NEPC is now available (88). Long noncoding RNA small nucleolar RNA host gene 3 (SNHG3) is expressed at high levels in prostate cancer tissues.SNHG3 mediates prostate cancer metastasis and progression by regulating TRIM25 through the sponge miR-487a-3p.SNHG3 expression and miR-487a-3p inhibitors can promote cell viability in prostate cancer (89). Targeting SNHG3 may help to prevent prostate cancer metastasis as well as the treatment of metastatic prostate cancer, for which research experiments are currently underway. In addition, relevant experiments have demonstrated that the expression of LncRNA-PRRT3-AS1 was significantly increased in prostate cancer cells, and the targeting relationship between lncRNA-PRRT3-AS1 and PPARN was demonstrated. PPARn expression was increased by targeting lncRNA-PRRT3-AS1, which inhibited the activation of the mTOR signaling pathway and thus inhibited the progression of prostate cancer cells. Therefore, targeting lncRNA-PRRT3-AS1 can also play an active role in the treatment of prostate cancer (90). In conclusion, lncRNAs have the potential to serve as non-invasive biomarkers and the ability to target therapies against tNEPC/NEPC, and they deserve to be tested in a clinical trial setting. In conclusion, lncRNAs have the potential to serve as non-invasive biomarkers and the ability to target therapies against tNEPC/NEPC, and they deserve to be tested in a clinical trial setting.

**Table 3:** Ongoing clinical trials against different targets for prostate cancer.

Clinical Trial ID	Target Point	Phase	Enrollment	Completion Date	Reference
NCT01799278	MYCN-AURKA	II	60	2018.01	(73)
NCT00676650	FOXA2-KIT	III	873	2013.02	(74)
NCT04702737	AMG757	I	–	2024.03	(78)
NCT01468532	SSTR 5-AS1	I	18	2021.03	(88)
Y20180831	SNHG3	–	50	–	(89)

Finally, nanotherapeutic systems may be the future direction of development of anticancer drugs. Some researchers have utilized nanomaterials as a carrier, loaded with si-lncRNA, to construct a nanotherapeutic system (si-LNC@PB) targeting prostate cancer cells. The nanotherapeutic delivery system has the advantages of precise drug delivery, high stability, and improved controlled release effect, which can effectively enhance the efficacy of prostate cancer treatment (91–94). This is a specific direction that needs further research.

## 7 Conclusion & future perspective

The molecular typing of NEPC is gradually demonstrated as the molecular pathologic typing of prostate cancer has been increasingly studied. Multiple transcription factors drive neuroendocrine carcinoma, and molecular typing of small cell carcinoma of the lung based on the transcription factors ASCL1, NEUROD1, POU2F3, and YAP1 can provide a basis for precision targeted therapy for lung cancer patients (95). NEPC has more similarities with small cell carcinoma of the lung, but due to the small number of clinical NEPC tissue samples, multicenter cooperation is needed to conduct research related to molecular typing, which is also a challenge. We also need to conduct further research to establish molecular typing of NEPC, identify the drivers and underlying factors of the disease, and formulate corresponding therapeutic regimens, which can help to realize the precision targeted therapy of NEPC. With the development of high-throughput sequencing technologies (e.g., RNA-seq and microarrays), it has been found that noncoding RNAs, especially lncRNAs, play an essential role in the process of tumorigenesis and progression, instead of being considered as genomic “enomic or “rnomicr”. Combining morphological and multi-omics techniques with artificial intelligence to predict the evolutionary outcomes associated with prostate cancer and propose targeted intervention strategies to delay or reverse the transformation of adenocarcinoma to neuroendocrine carcinoma is a necessary intervention to prolong and enhance the quality of patient survival. Aberrant expression of lncRNAs, an indispensable component of the human transcriptome, is expected to be developed as a biomarker for interfering with NEPC formation and enzalutamide resistance. However, despite the large amount of experimental data suggesting that lncRNAs are promising for treating enzalutamide resistance, their clinical translation is still an urgent problem that needs to be solved. Furthermore, multiple resistance mechanisms can coexist due to the complexity of resistance, limiting the precision of a single therapy. Although challenging, the challenges facing enzalutamide resistance will eventually be overcome as research in preclinical work deepens.
